# Cost-effectiveness analysis of sarcopenia management interventions in Iran

**DOI:** 10.1186/s12889-023-15693-w

**Published:** 2023-05-04

**Authors:** Ali Darvishi, Gita Shafiee, Narges Zargar Balajam, Mohsen Rezaei Hemami, Navid Ostovar, Ramin Heshmat

**Affiliations:** 1 Chronic Diseases Research Center, Endocrinology and Metabolism Population Sciences Institute, Tehran University of Medical Sciences, NO 10, Jalale-Al-Ahmad Ave, Chamran Highway, Tehran, 1411713137 Iran; 2 Department of Health Management and Economics, School of Public Health, Tehran University of Medical Sciences, Tehran, Iran; 3 Endocrinology and Metabolism Research Center, Endocrinology and Metabolism Population Sciences Institute, Tehran University of Medical Sciences, Tehran, Iran; 4 Perspectum Ltd, Oxford, UK

**Keywords:** Sarcopenia, Management intervention, Economic evaluation, Cost-Utility Analysis (CUA), Quality-adjusted life years (QALYs)

## Abstract

**Objectives:**

Identification the optimal management intervention of sarcopenia is a concern of health systems. We aimed to analyze the cost-effectiveness of sarcopenia management strategies in Iran.

**Methods:**

We constructed a lifetime Markov model based on natural history. The strategies comparedincluded exercise training, nutritional supplements, whole body vibration (WBV), and various exercise interventions and nutritional supplement combinations. A total of 7 strategies was evaluated in addition to the non-intervention strategy. Parameter values were extracted from primary data and the literature, and the costs and Quality-adjusted life years (QALYs) were calculated for each strategy. Deterministic and probabilistic sensitivity analysis, including the expected value of perfect information (EVPI), was also performed to determine the robustness of the model. Analyses were performed using the 2020 version of TreeAge Pro software.

**Results:**

All seven strategies increased lifetime effectiveness (QALYs). The protein and Vitamin D_3_ (P + D) strategy had the highest effectiveness values among all strategies. After removing the dominated strategies, the estimated ICER for the P + D compared to Vitamin D_3_ alone (D) strategy was calculated as $131,229. Considering the cost-effectiveness threshold ($25,249), base-case results indicated that the D strategy was the most cost-effective strategy in this evaluation. Sensitivity analysis of model parameters also demonstrated the robustness of results. Also, EVPI was estimated at $273.

**Conclusions:**

Study results, as the first economic evaluation of sarcopenia management interventions, showed that despite the higher effectiveness of D + P, the D strategy was the most cost-effective. Completing clinical evidence of various intervention options can lead to more accurate results in the future.

**Supplementary Information:**

The online version contains supplementary material available at 10.1186/s12889-023-15693-w.

## Introduction

Sarcopenia is a disease characterized by the progressive loss of skeletal muscle mass and strength with the risk of increased physical disability, falls, fractures, reduced quality of life, and death [1–3]. Between 40 and 80 years, the decline muscle mass occurs as much as 30–50%, so aging is the main cause of sarcopenia [4–6].

The updated definition of sarcopenia by the European Working Group on Sarcopenia in Older People (EWGSOP-2) has specified criteria, including muscle strength, muscle mass, and physical performance [7]. According to the EWGSOP-2, the highest prevalence of sarcopenia has been observed in Oceania and the lowest in Europe. This prevalence range varies from 10 to 27% in individuals over 60 years. Regarding EWGSOP-2 determination, men demonstrate a higher prevalence of sarcopenia than women [8, 9].

Although few studies have evaluated the prevalence of sarcopenia in Iran [10, 11], in an overview study among the elderly above 59 years old in Tehran health centers, the prevalence rate using various definitions of the Asian Working Group for Sarcopenia (AWGOS) and EWGOSOP was reported at 16.5% and 32.5%, respectively [12]. The Bushehr Elderly Health (BEH) program found that using EWGSOP-1 criteria, the prevalence of sarcopenia was 19.7% in males and 13.6% in females. Using EWGSOP-2 measures, these values were estimated to be 12.7% and 5.42% in men and women, respectively [13].

Predictions have revealed that sarcopenia, which threatens personal health and reducing life satisfaction, also imposes a heavy economic burden on healthcare systems. In the USA, the healthcare charge for sarcopenia in 2000 was determined to be $18.5 billion (almost 1.5% of total healthcare costs) [14]. Furthermore, hospitalization expenditure has been found to increase by up to 34% among patients ≥ 65 years with sarcopenia [15]. £2.5 billion extra annual cost is estimated for health and social care provision in British people aged 71–80 with sarcopenia and muscle weakness [16].

Considering the prevalence of sarcopenia as a major global public health problem, finding a solution for the prevention and relative recovery of this disease is a concern of many societies. Therefore, the first step is to investigate operative interventions in handling sarcopenia. Due to the lack of effectiveness of medicines on sarcopenia patients, non-pharmacological interventions with more effect were considered a better alternative to diminishing the speed of morbidity progression [17]. Early interventions are the principal strategy to improve conditions in elderly people with sarcopenia [18].

One of the main causes of sarcopenia can be attributed to an inactive lifestyle. Inertia and not using muscles will cause their atrophy. Physical exercise, consisting of resistance and strength exercises, can be promising for reducing the loss of muscle mass and strength caused by aging [19, 20]. However, these routine exercises may not be suitable for all frail or aging people with physical limitations and worsen their conditions. New research found vibration therapy (VT) to be a safe and effective alternative for improving and maintaining muscle mass in these people [21, 22].

A diet that does not provide enough calories and protein leads to a loss of muscle mass. Branched-chain amino acids such as Leucine play a significant role in the formation of muscle proteins. Thus, based on the positive effects of protein on muscle systems, it has been investigated as a hypothetical therapeutic intervention for sarcopenia [23, 24]. Recent studies suggest that vitamin D can be considered an evidence-based treatment for sarcopenia due to its ability to inhibit myostatin expression in muscle tissue and protect skeletal muscle against acute damage [25, 26].

There are no standard guidelines for the control and treatment of this disease. Therefore, it is important to use rational interventions for mature patients with sarcopenia to ameliorate their quality of life. Also, some investigations have examined several of these interventions together [27, 28].

Since this disease endures huge costs for society, and on the other hand, the increase in average life expectancy and the subsequent rise in the elderly population have caused a momentous prevalence of sarcopenia, so the cost-effective management of this disease has become particularly important. Therefore, we aimed to evaluate management interventions in terms of cost-effectiveness. Identifying the optimal options for managing this disease could reduce the economic burden it causes in the community.

## Materials and methods

In a full economic evaluation, a cost-utility analysis (CUA) was performed to evaluate sarcopenia management interventions based on Iran's health system perspective. This evaluation collected desired evidence based on sarcopenic patients aged ≥ 60.

Various strategies are used for the treatment and management of sarcopenia, although in many cases, there is no evidence of their efficacy from a clinical point of view. Only strategies that showed significant efficacy based on the latest systematic review studies were included in the comparative evaluation in this study. The strategies compared in this evaluation are presented in Table 1. This table also provides a detailed description of the interventions, including dosage and duration.

**Table 1:** Strategies, dosage and duration

Strategy	Dosage and Duration
Exercise (E)	3 training sessions of 1.5 h per week
Protein supplementation (P)	Whey protein 45 g/day
Vitamin D3 (D)	Vitamin D 800 IU/day
Whole body vibration (WBV)	3 sessions per week
Protein supplementation and Vitamin D3 (P + D)	(Vitamin D 800 IU/day + Whey protein 45 g/day)
Vitamin D3 and Exercise (D + E)	(Vitamin D 800 IU/day + 3 training sessions of 1.5 h per week)
Protein supplementation, Vitamin D3, and Exercise (P + D + E)	(Whey protein 45 g/day + Vitamin D 800 IU/day + 3 training sessions of 1.5 h per week)

### Modeling

The model was designed to reflect the natural course of sarcopenia, as well as the nature and effectiveness of interventions, intermediate outcomes, and associated costs. According to the findings regarding the clinical evidence of the disease and the effectiveness of the interventions and expert opinions, the risk of falling and consequently the risk of fracture, the mortality risk, and the reduction in the level of health-related quality of life (HRQoL) were considered as the main outcomes of the interventions.

**Fig. 1:**
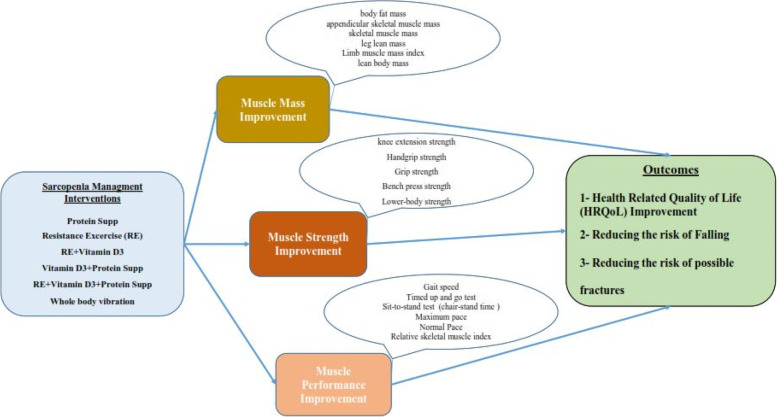
Sarcopenia management conceptual model

The main clinical outcome of the model was the risk of falling, and the effectiveness of the interventions was also included in the model in the form of reducing the risk of falling, which is considered through improvement in the three main indicators of sarcopenia. In the model, the effectiveness of the interventions is first considered on three indicators of muscle mass, muscle strength, and physical performance. Considering that the change in each of these three indicators is related to the change in the risk of falling, the improvement of each of these indicators is modeled by reducing the probability of falling. The conceptual model of the study regarding the effectiveness of interventions can be seen in Fig. 1.

**Fig. 2:**
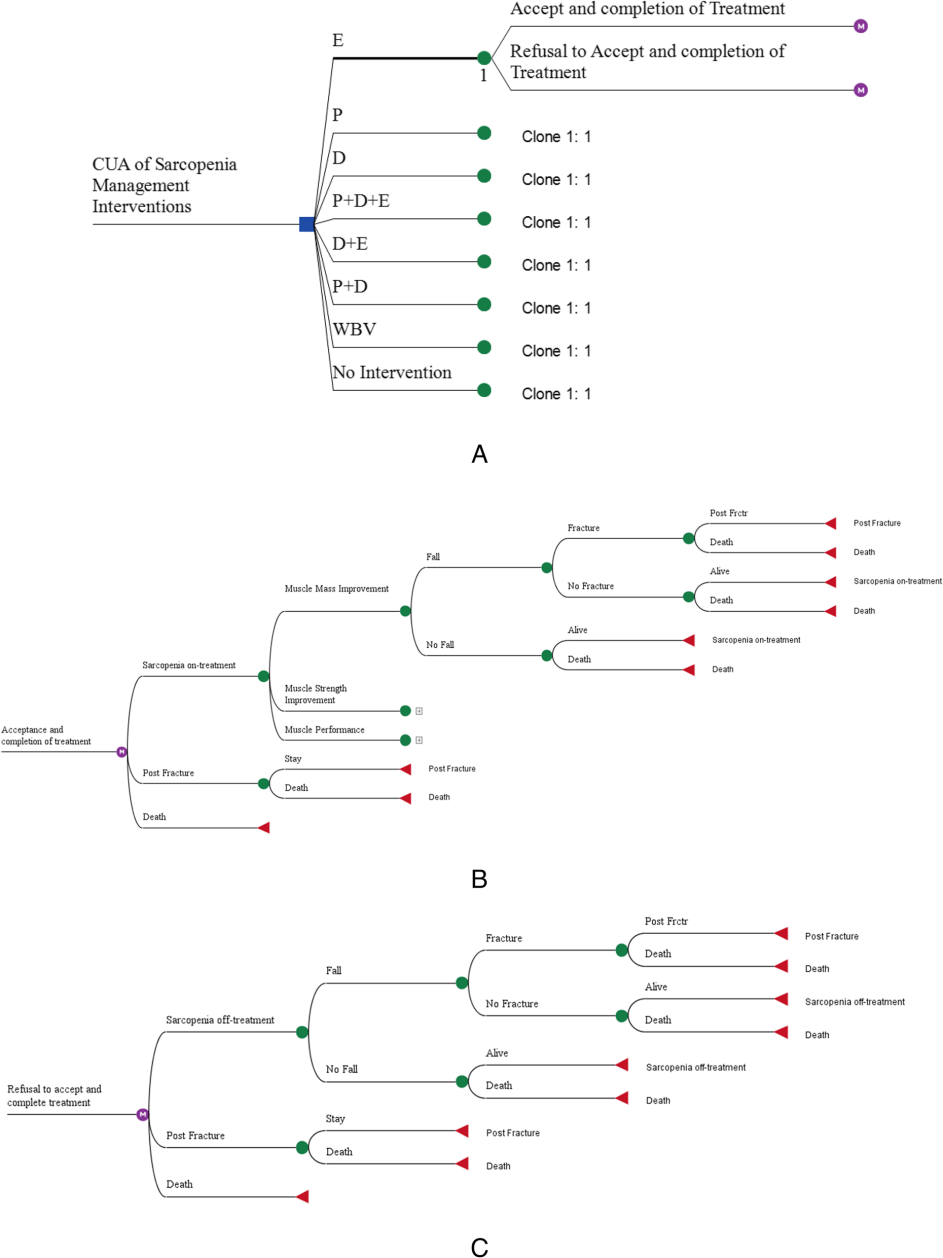
Decision tree and Markov model structures of CUA of sarcopenia management strategies

The structure of the cost-utility model begins with a decision tree, in such a way that sarcopenic patients are categorized into two groups: patients who accept the intervention and adhere during the treatment period, and patients who do not accept the intervention and are not treated. Patients are then entered into discrete Markov structures (Fig. 2-a). The first Markov structure is related to the individuals who accept the intervention (On-treatment) (Fig. 2-b), and the second Markov structure is related to the individuals who do not accept the intervention or who do not adhere (Off-treatment) (Fig. 2-c). Also, in the strategy of no intervention, individuals are modeled based on this Markov structure (Off-treatment).

The model in each strategy consists of three Markov health states, including the state of sarcopenic patients (On-treatment and Off-treatment), post-fracture health state, and death. The initial distribution is that all individuals are Sarcopenic patients On-treatment and Sarcopenic patients Off-treatment in the first cycle, in the group of patients who accept the intervention and the group who do not accept the intervention, respectively. During the Markov simulation, individuals are transitioned according to the transition probabilities between these states, so that at the end of each cycle, people either remain in the same health state or are transferred to other health states. At the end of the final cycle, all individuals are in the death state. It should be noted that fracture is considered an event after falling. Since the Health-related quality-of-life (HRQoL) and the costs of fracture during the first year of the occurrence of a fracture are different from the years, individuals who face a fracture and survive will enter the state of post-fracture in the next cycle.

It should be noted that comorbidities have been omitted, due to the simplification of the model and evaluation. The model structure and events process are similar in all strategies, and only the parameter values are different. The Markov structure can be seen in two groups of On-treatment and Off-treatment in Fig. 2.

### Model assumptions

As mentioned, at the beginning of the model, individuals were divided into two groups: those who accepted treatment intervention and those who did not. This division was done according to the importance of patients' acceptance and adherence to the treatment because of treatment effectiveness. However, since quantitative evidence in this regard were not available for the compared interventions, 90% of treatment acceptance and adherence were considered in the model in all interventions. Based on this, the assumption of the model is that the patients who do not accept the intervention or do not have continuity in the treatment are modeled in the group of patients off-treatment. Considering this part in the modeling is because, considering the nature of the interventions for the effectiveness of the treatment, acceptance and persistence in the treatment by patients in this age group is very important.

The effectiveness of interventions was considered by three indicators of muscle mass, muscle strength, and muscle function. Since quantitative evidence regarding the difference in the effectiveness of the interventions on the three mentioned indicators was not available, the effectiveness of the treatment was equally weighted.

The model time horizon was considered a lifetime. Also, the Markov cycle length was considered one year due to the nature of changes in disease health states.

In this model, fall and Fracture were considered events in life cycles. Due to this issue, people have a lower quality of life after falls and fractures and bear additional treatment costs.

### Extraction of parameter values

The study's final outcome was quality-adjusted life years (QALY), which was modeled according to the disutility caused by events and the utility values in health states. Each strategy was ultimately evaluated based on cost per QALY. The evidence related to the disutility of the occurrence of events and the degree of utility in each health state had been extracted from literature [29, 30].

According to the evaluation perspective, we considered only direct costs, and indirect costs were not included in the analysis. The costs of each strategy, including the cost of interventions in each cycle, were calculated based on the cost unit used in each intervention, treatment periods, and the number of repetitions. For this purpose, the data collected from the evidence related to the effectiveness was used [28, 31] as expert opinions. Also, other treatment costs, including the cost of periodical visits and the cost of laboratory tests, were calculated based on the treatment protocols, consultation with the specialized medical team, and official tariffs. Based on this, an average of two physician visits per year and of annual routine laboratory tests were considered, and costing was done based on official tariffs. These costs are considered for all modeled individuals in the treated group. The cost of fracture and post fracture treatment was taken beyond past Iranian studies and adjusted based on the 2022 prices [32]. The cost of other possible treatment and supportive care was not taken into account in the cost calculations due to the fact that it is different and variable based on the condition of different patients as well as other underlying diseases. Naturally, the effectiveness of these measures is not included in the model.

Based on the Food and Drug Organization website, the unit cost of vitamin D3 interventions (Vitamin D 800 IU/day) was calculated based on the average price of the brands available in Iran. The cost of protein supplements was based on the pricing of different brands of Whey protein and calculated based on 45 g/day dosage. The cost of exercises was calculated according to the pricing of the monthly costs of fitness clubs and rehabilitation centers in Tehran, Iran. The period and time intervals of stretching and resistance exercises under the supervision of the trainer were 3 sessions of 1.5 h per week. The unit cost calculation of WBV was also based on the pricing from rehabilitation centers in Tehran, and finally, their average was calculated. The minimum and maximum calculation values were also considered in the sensitivity analysis of the model. The parameter values can be seen in Table 2.

**Table 2:** Model input and parameters

Statistic variable	Base case	SD/(CI)	Distribution	Source
Time Horizon	Lifetime			
Annual discount rate (Costs)	0.05	(0.02–0.1)	Beta	
Annual discount rate (Outcomes)	0.05	(0.03–0.08)	Beta	
Probability of death in non-sarcopenic individuals (Normal Pop 60 yrs)	0.0095			IRI Life Table
The hazard ratio of Sarcopenia Mortality	1.6	(1.24–2.06)	Log-Normal	[33]
Relative Risk of Fracture Mortality (1^st^ yr)	6.57	(5.54–7.29)	Log-Normal	[34]
Probability of falling in Sarcopenic individuals	0.155	± 0.041	Beta	[35]
Probability of fracture in Sarcopenic individuals	0.33	± 0.06	Beta	[35]
*Fall Risk Reduction (MM Improvement) %*				
P	3.64	± 0.91	Beta	[28, 36]
E	14	± 3.5	Beta	[28, 36]
P + D	46.52	± 11.63	Beta	[28, 36]
P + D + E	24.35	± 6.0875	Beta	[28, 36]
D + E	15.31	± 3.8275	Beta	[28, 36]
*Fall Risk Reduction (MS Improvement) %*				
WBV	1.15	± 0.2875	Beta	[31, 37]
P	1.6	± 0.4	Beta	[28, 37]
P + D	1.6	± 0.4	Beta	[28, 37]
P + D + E	3.85	± 0.962	Beta	[28, 37]
D + E	3.7	± 0.925	Beta	[28, 37]
*Fall Risk Reduction (MP Improvement) %*				
WBV	33.23	± 8.3075	Beta	[31, 36]
P	8.82	± 2.205	Beta	[28, 36]
P + D	20.1	± 5.025	Beta	[28, 36]
P + D + E	37.33	± 9.332	Beta	[28, 36]
D	27.08	± 6.77	Beta	[28, 36]
*The annual average cost of treatment interventions ($)*				
P	5463.329	± 1515.555	Gamma	Our study
E	4268.785	± 853.75	Gamma	Our study
P + D	5592.605	± 1536.569	Gamma	Our study
P + D + E	9861.39	± 7477.532	Gamma	Our study
D + E	4398.06	± 1662.537	Gamma	Our study
WBV	7126.312	± 1790.565	Gamma	Our study
Cost of treatment and care of fractures (1^st^ yr)	12,588.214	± 3147.053	Gamma	[32] and calibration
The annual cost of treatment and care of post-fracture state	2517.64	± 503	Gamma	[32] and calibration
Annual Cost of Visits and Laboratory Tests	400.409			Our study
Utilities				
Sarcopenic individuals (> 60 years)	0.785	± 0.1962	Beta	[29]
Disutility in 1^st^ year after Fracture	0.25	± 0.025	Beta	[30]
The disutility of fracture after 1^st^ year	0.17	± 0.017	Beta	[30]

*SD* Standard Deviation, *CI* Confidence Interval, *P* Protein, *D* Vitamin D_3_, *E* Exercise, *WBV* Whole body vibration

Other related parameters and variables include transition probabilities, the effectiveness of treatment on the three indicators of muscle mass, muscle strength, and muscle function, risks related to the occurrence of falls, fall Risk Reduction due to the improvement of the condition of the three indicators, fracture risk caused by falling and other parameters were extracted from internal and international evidence. In this regard, a separate search was conducted for each parameter based on specific keywords and strategies in scientific databases, studies that had relevant evidence were classified, and finally, the best available evidence was extracted. The details of the model parameter values ​​can be seen in Table 2.

A 5% discount rate for both costs and QALYs in the model was used in base-case analysis based on the Health Technology Assessment Office of Iran's Ministry of Health recommendation. Also, 2 to 10% and 3 to 8% was considered for sensitivity analysis discount-rate for costs and QALYs, respectively.

### Base-case and sensitivity analysis

To analyzing and determine the most cost-effective strategy, the Incremental cost-effectiveness ratio (ICER) index is used. The formula of this index is as follows:

$$\mathrm{ICER}=({\mathrm{C}}_{1}-{\mathrm{C}}_{2})/({\mathrm{E}}_{1}-{\mathrm{E}}_{2})$$

In this evaluation, the cost-effectiveness threshold (willingness to pay (WTP)) was considered equal to one times the Iran's GDP per capita in 2022, equivalent to 25,249 dollars (70 million Rials). Rial values in the present study were converted using the purchasing power parity (PPP) dollar conversion factor to Rial equal to 29704 Rials [38].

Due to the uncertainty regarding some parameters used in the model, deterministic and probabilistic sensitivity analyses were performed. First, one-way sensitivity analysis was performed using a tornado diagram for all deterministic uncertain parameters and estimated the effect of individual changes in parameter values on ICER results.

Also, taking into account the probabilistic distribution of some uncertain variables using Monte-Carlo simulation according to 1000 repetitions of simulation, probabilistic sensitivity analysis (PSA) was performed, and cost-effectiveness acceptability curve, ICE Scatter Plot and other reports were extracted.

The distributions used in the sensitivity analysis are as listed in Table 1. In cases where no evidence was found regarding the distribution of the desired variable, 10% to 30% of the average parameter was considered standard deviation, and depending on the variable type, the appropriate distribution was chosen.

Considering that some parameters in the model were associated with high uncertainty, the expected value of perfect information (EVPI) was also estimated based on the Monte-Carlo simulation results. The EVPI is the price a healthcare decision-maker is willing to pay to have perfect information about all factors influencing the preferred treatment choice due to a cost-effectiveness analysis [39].

The entire process of modeling, base-case analysis, and all stages of sensitivity analysis was carried out using version 2020 of the TreeAge software.

## Results

### Base-case analysis

The economic evaluation of seven sarcopenia strategies was performed with no intervention. Table 3 shows the results of the analysis of the overall strategies together, and Table 4 shows the results of the analysis of the strategies by excluding the Dominated strategies. D + P strategy had the highest effectiveness values (QALYs) among all strategies but compared with the D strategy in terms of cost-effectiveness, the estimated ICER for the P + D compared to D was calculated as $131,229. Comparison to the cost-effectiveness threshold ($25,249) indicated the lack of cost-effectiveness of P + D. So, the D strategy was a cost-effective intervention in this evaluation. Additionally, Figure S1 in Supplementary Information also shows the results of the base-case analysis in the form of a cost-effectiveness plane.

**Table 3:** Base case CUA of Sarcopenia Management Strategies in Iran (All referencing common baseline)

Strategy	Cost($)	Incremental Cost($)	QALYs	Incremental QALYs	ICER ($/QALY)	Category
D	13,458.63		8.34			undominated
No Intervention	13,460.61	1.98	8.13	-0.20	-9.86	Dominated
E	48,200.20	34,741.57	8.24	-0.10	-347,064.17	Dominated
D + E	49,551.44	36,092.81	8.30	-0.03	-1,058,670.22	Dominated
P	58,159.21	44,700.57	8.25	-0.08	-509,894.1	Dominated
P + D	62,362.63	48,904	8.71	0.37	131,229.95	undominated
WBV	735,551.9	60,093.26	8.40	0.06	884,405.46	Dominated
P + D + E	101,984.47	88,525.84	8.70	0.36	240,237.77	Dominated

*ICER* Incremental cost-effectiveness ratio, *QALY* Quality adjusted life year, *P* Protein, *D* Vitamin D_3_, *E* Exercise, *WBV* Whole body vibration

**Table 4:** Base case CUA of Sarcopenia Management Strategies in Iran (Excluding Dominated)

Strategy	Cost($)	Incremental Cost($)	QALYs	Incremental QALYs	ICER ($/QALY)
D	13,458.63		8.34		
P + D	62,362.63	48,904	8.71	0.37	131,229.95

*ICER* Incremental cost-effectiveness ratio, *QALY* Quality adjusted life year, *P* Protein, *D* Vitamin D_3_, *E* Exercise, *WBV* Whole body vibration

### Sensitivity analysis

#### Deterministic Sensitivity Analysis (DSA)

**Fig. 3:**
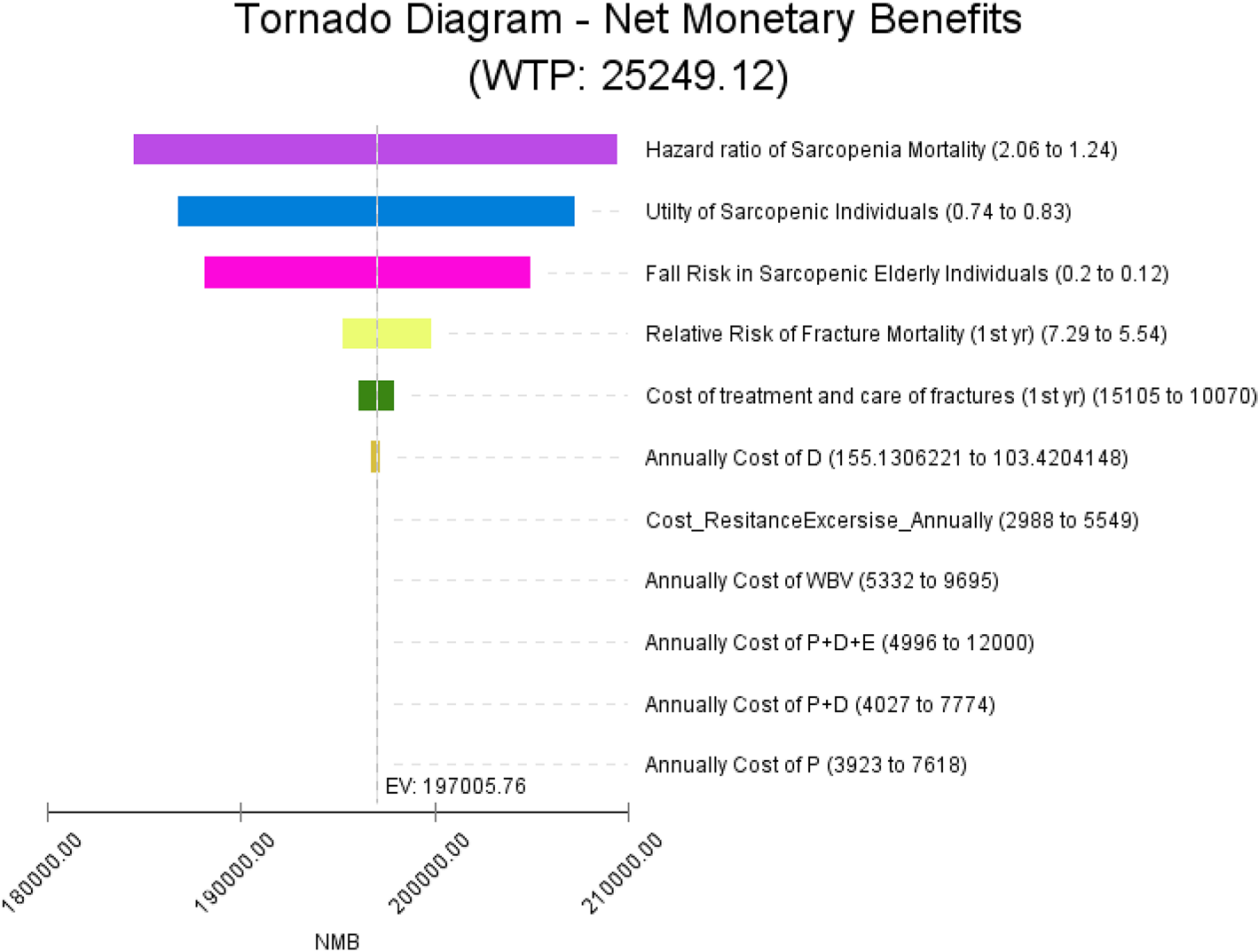
Tornado diagram of cost-utility analysis of sarcopenia management strategies. *P: Protein; D: Vitamin D*_*3*_*; E: Exercise; WBV: Whole body vibration*

Tornado diagram was extracted based on the net monetary benefits (NMBs) (Fig. 3). As can be seen, changing the values of the HR of sarcopenia mortality and the utility of the sarcopenic individuals had the greatest effect on the changes of the NMBs, but none of the variables at any point led to a change in the overall results of the analysis.

#### Probabilistic Sensitivity Analysis (PSA)

**Fig. 4:**
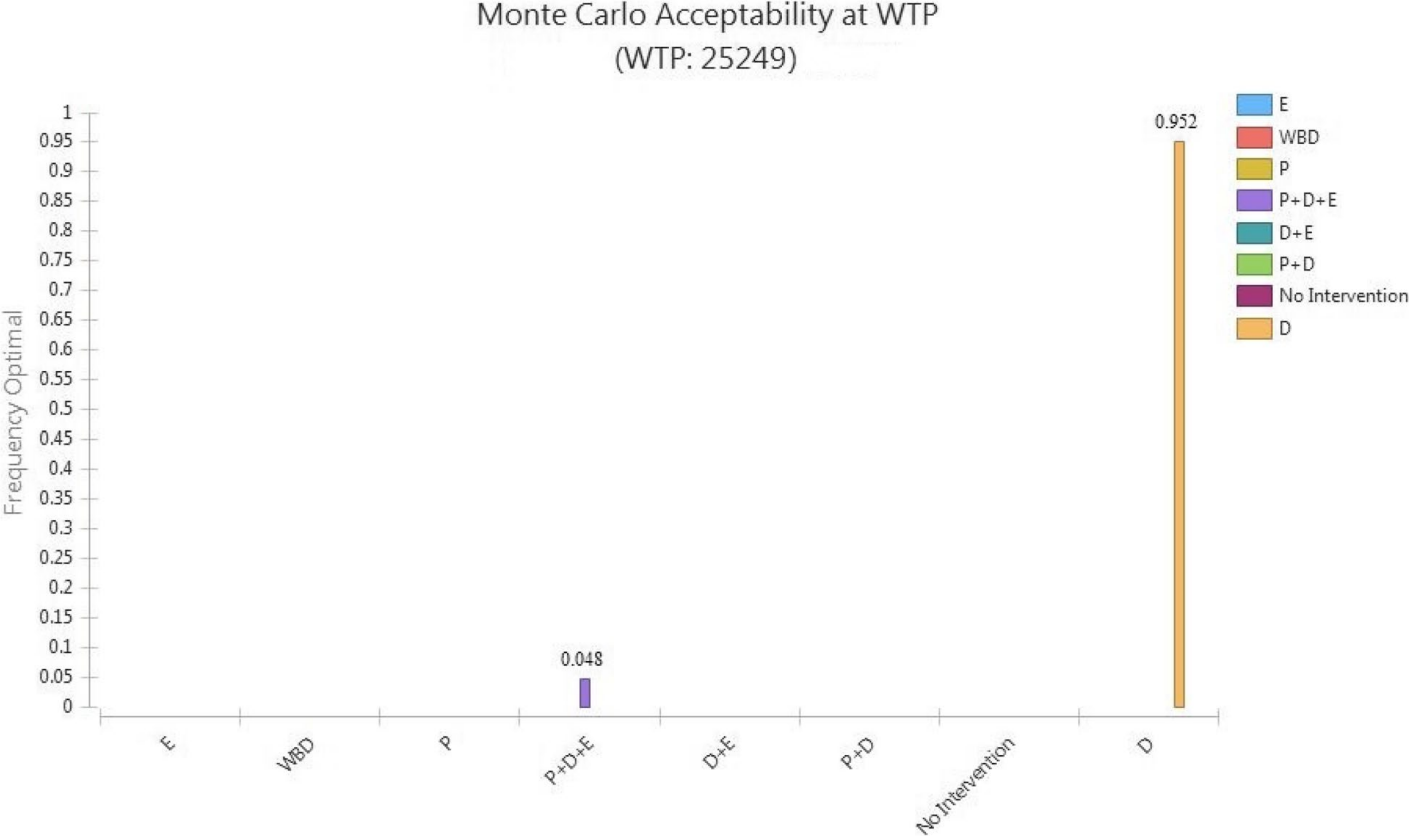
Monte-carlo Simulation (Acceptability at WTP) of cost-utility analysis of sarcopenia management strategies. *P: Protein; D: Vitamin D*_*3*_*; E: Exercise; WBV: Whole body vibration*

PSA was performed by considering the distribution function of some model uncertain parameters, using Monte-Carlo simulation, by1000 repetitions of the simulation. Figure 4 shows the probability of optimality for each strategy. The cost-effectiveness probability of the D strategy was 95%. Moreover, the probability of optimality of the P + D + E was equal to 5% and while for all other strategies was zero.

Also, incremental cost-effectiveness scatter plot of the P + D strategy compared to D can be seen in the supplementary information in this regard (Figure S2).

**Fig. 5:**
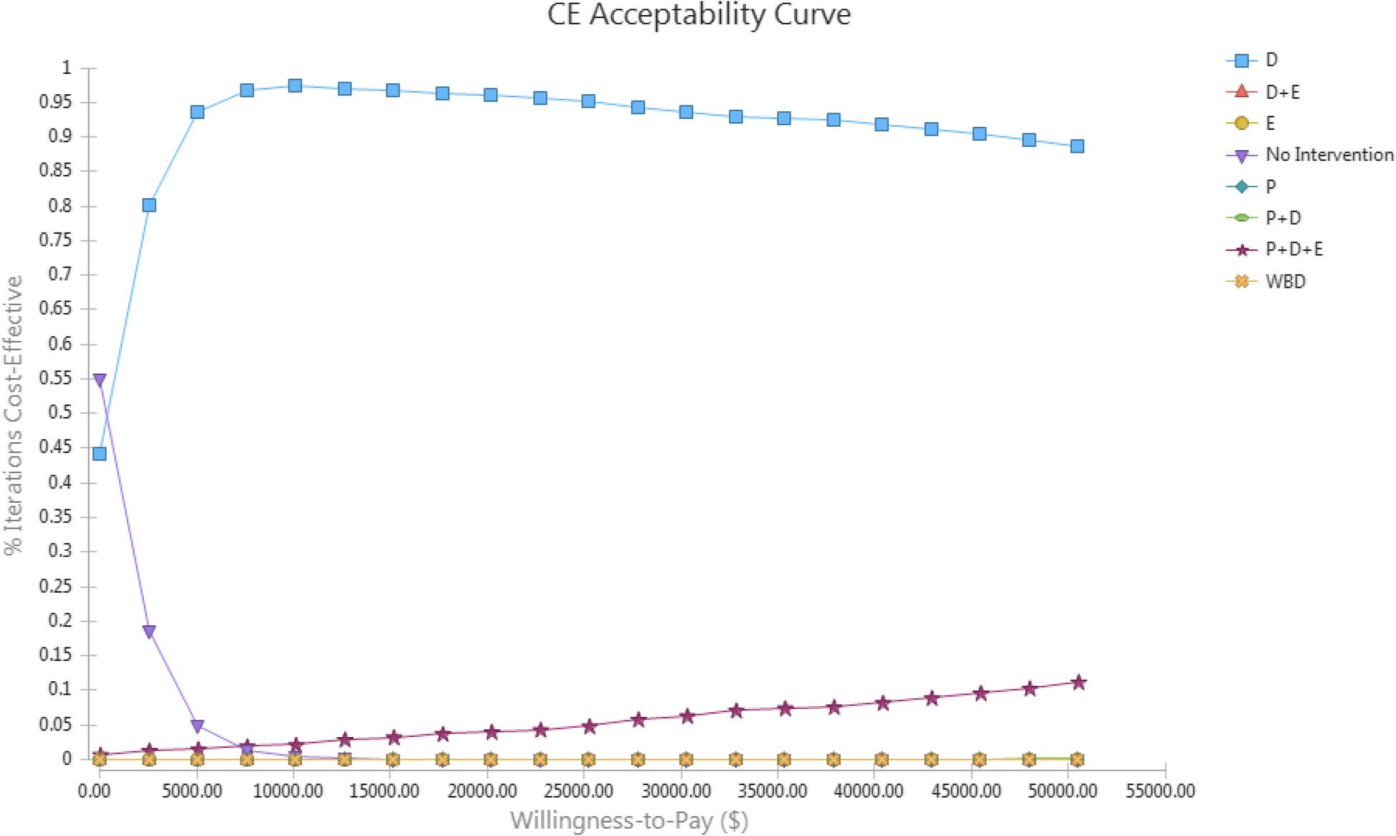
Monte-carlo simulation (Cost Effectiveness Acceptability Plane) of cost-utility analysis of sarcopenia management strategies. *P: Protein; D: Vitamin D3; E: Exercise; WBV: Whole body vibration*

Figure 5 also shows the CostEffectiveness Acceptability Curve. As can be seen, with the increase in the cost-effectiveness threshold value, the probability of the cost-effectiveness of the P + D + E strategy increases and reaches up to 15% at approximately twice the threshold value. Similarly, the cost-effectiveness of the D strategy decreases.

In general, the results of DSA and PSA showed that the base-case analysis results are robust with a high probability.

#### EVPI

Monte-Carlo simulation results regarding the EVPI can be seen in Table 5. Based on this, the average incremental cost in perfect information was estimated to be $186, and the average incremental effectiveness with complete information is estimated to be 0.018 QALY units. Finally, the EVPI is estimated at $273.

**Table 5:** Monte-carlo simulation (EVPI) of cost-utility analysis of sarcopenia management strategies

Variable	Value
Weight on Eff. (WTP)	25,249.13
EVPI\EVPPI (Incr. NMB)	273.62
Avg. Incremental. Cost with Perfect Info	186.61
Avg. Incremental. Eff with Perfect Info	0.018
Optimal Strategy	8

*WTP* Willingness to pay, *EVPI* expected value of perfect information, *EVPPI* Expected Value of Partially Perfect Information, *NMB* Net monetary benefit

**Fig. 6:**
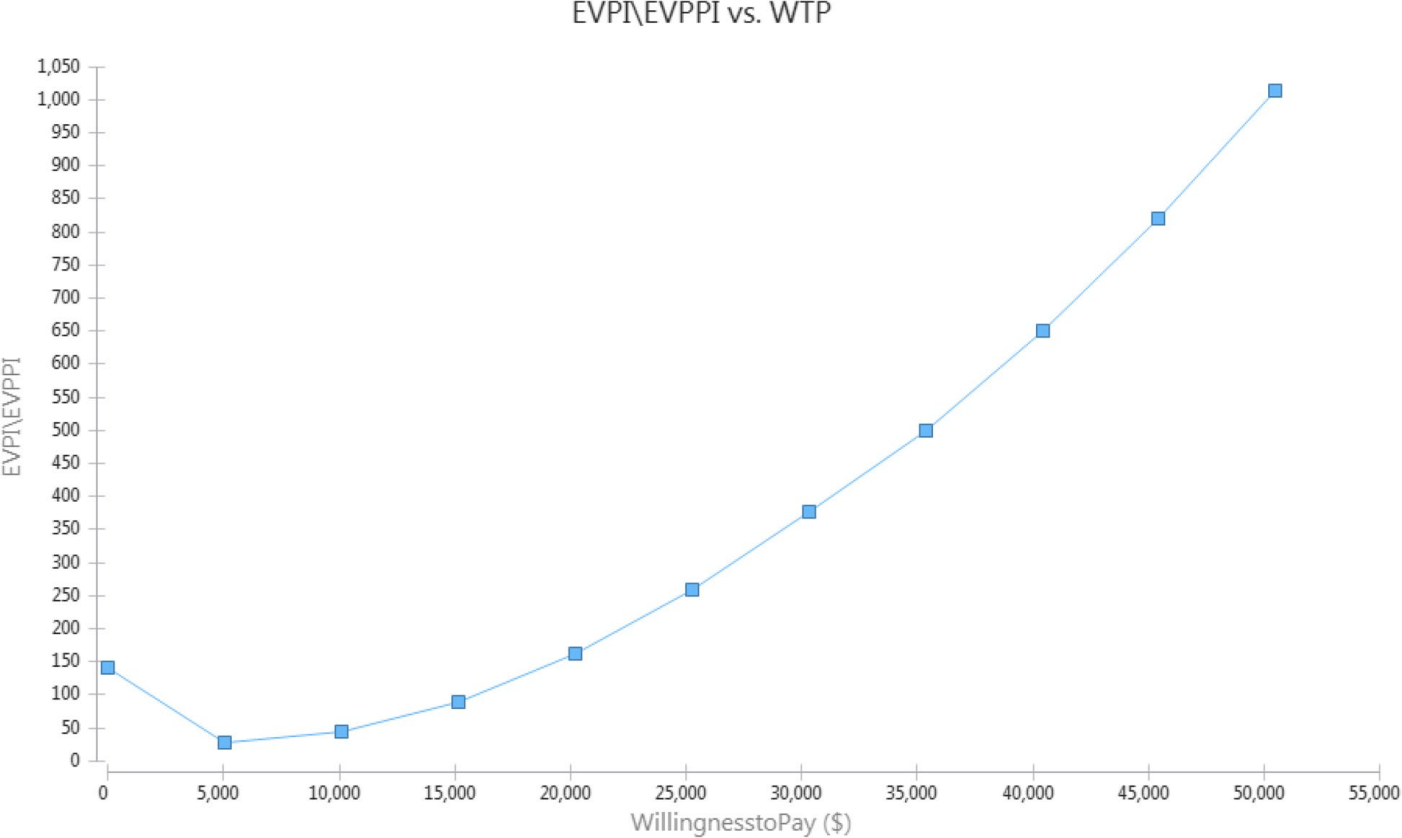
Monte-carlo Simulation (EVPI) of cost-utility analysis of sarcopenia management strategies in Iran. WTP: Willingness to pay; EVPI: expected value of perfect information; EVPPI: Expected Value of Partially Perfect Information

Figure 6 also shows the amount of EVPI at different cost-effectiveness thresholds, which naturally increase with the threshold increase.

## Discussion

Considering the growing trend of aging in the world, it is essential to pay attention to age-related diseases, so that with timely diagnosis and treatment of diseases, we can move in the direction of reducing the overall costs of the health system and improving the people's quality of life. As a disease of old age, sarcopenia will be great importance, and it is important to use the most effective management strategies to minimize the possible consequences and significant financial burden. According to a study, the direct costs of sarcopenia were estimated at about 1.5% of the total annual health costs in the United States [40].

A comparison of different management strategies for sarcopenia was conducted from the perspective of Iran's health system. Because of the little comprehensive evidence regarding the clinical effectiveness of sarcopenia interventions, in this study, the research steps were designed and implemented in a systematic and step-by-step manner based on the best available evidence.

Base-case results indicated that vitamin D was the cost-effective strategy. Other combination strategies with vitamin D, including vitamin D and protein (P + D), vitamin D and protein along with exercise (P + D + E), and vitamin D along with exercise (D + E), despite the higher effectiveness and the increase in the amount of QALYs obtained, according to Iran's cost-effectiveness threshold were not cost-effective. Regarding the P + D and D strategies, the two undominated options in this evaluation, the comparative results showed that the estimated ICER of P + D compared to the D strategy was approximately five times more than the cost-effectiveness threshold.

In this regard, it can be said that although sarcopenia interventions lead to an increase in QALY values during the lifetime, this increase is not substantial. For this reason, only interventions that can be done at a relatively low cost will be cost-effective. Of course, these results have been obtained according to the available evidence regarding the effectiveness of interventions. According to a systematic review and network meta-analysis conducted by Cheng et al. (2021), incorporating vitamin D into a standard treatment regimen for sarcopenia could potentially aid in the restoration of function. The study suggests that administering vitamin D supplements to individuals with sarcopenia may result in a notable improvement in grip strength, particularly when combined with exercise and protein supplementation [28]. Current evidence for supplementation with vitamin D alone is not robust, while vitamin D in patients with sarcopenia could be a routine supplementation due to safety and not high cost. Other previous studies have shown that vitamin D plays an important role in maintaining muscle health and preventing sarcopenia in older adults [41, 42]. A systematic review and meta-analysis of randomized controlled trials also found that vitamin D supplementation improved muscle strength and function in older adults [43]. This evidence supports the cost-effectiveness of vitamin D as a management strategy for sarcopenia. More complete and accurate evidence in this regard in the future yield different resultsdifferent results.

The results of DSA and PSA also completely confirm the robustness of base-case results. In this regard, in the considered cost-effectiveness threshold, the Monte-Carlo simulation showed that the probability of the cost-effectiveness of the D strategy alone is more than 95%. The only other strategy with an optimal chance was in this simulation the P + D + E with less than a 5% chance of cost-effectiveness.

In this study, the EVPI was also calculated. According to the simulation, EVPI was estimated to be $273. This means that at the desired cost-effectiveness threshold, the expected value of obtaining perfect information about the uncertainty of some model parameters was $273. This amount increased with the increase of cost-effectiveness threshold limits. Generally, obtaining perfect information can help identify the most appropriate treatment option with higher certainty [39].

Based on our knowledge, the evaluation carried out in the present study was the first economic evaluation regarding sarcopenia management interventions so far. Previously, in general, only one economic evaluation was done in the field of sarcopenia disease, which evaluated screening methods for this disease [44]. Other economic studies have focused on the costs of disease, such as the study by Janssen et al. (2004) and Goates et al. (2019) in the United States [40, 45] and the study by Sousa et al. (2016) in Portugal [15].

In this evaluation, according to the available evidence, increasing the HRQoL, reducing the risk of falling, reducing the risk of fracture, and reducing the probability of death were considered outcomes of disease management interventions, and other possible outcomes were ignored due to the limitations of the evidence.

In this evaluation, a combination of supplements, exercise, and WBV was considered, and the medicines that were sometimes prescribed to treat sarcopenic patients were not included in the study. In this regard, it should be noted that a particular medicine for treating and reducing sarcopenia complications has not yet been approved. On the other hand, our investigations from review studies showed that no significant efficacy of the medicines investigated was reported [46–48].

As mentioned in the methods section, at the beginning of the structure of the decision analysis model, patients were divided into two groups, treatment acceptance and non-treatment acceptance. However, due to the lack of evidence in this regard separately for the compared interventions, the probability of treatment acceptance were assumed to be 90% in the model for all interventions. However, considering this division at the beginning of the model was only because of drawing attention to the importance of the difference in treatment acceptance and adherence in different interventions on the results of economic evaluation. In such a way, it was likely that interventions with exercises had a lower acceptance and adherence rate among patients at elderly ages. This problem also shows the importance of producing evidence in this regard.

Also, in this study, the initial effectiveness of the interventions was considered on the three indicators of muscle mass, muscle strength, and muscle function. Considering that quantitative evidence regarding the difference in the effectiveness of the interventions on the three mentioned indicators was not available, the effectiveness of the treatment was weighted equally. This is another limitation of the present study.

Generally, by expanding the evidence and providing more access to the values of the required parameters, and removing the limitations of the study, more complete models can be designed and implemented in the future to identify the most cost-effective strategies in the management of sarcopenia.

## Conclusion

The base-case and sensitivity analysis results showed that despite the higher effectiveness of vitamin D and protein supplementation, the vitamin D alone strategy was highly cost-effective in managing sarcopenia in the Iranian population. This could have practical implications for healthcare providers and policymakers in Iran, who could consider implementing a vitamin D supplementation program as part of their approach to managing sarcopenia. This can assist in the efficient allocation of resources and improve the overall management of sarcopenia in the population. Additionally, the study highlights the importance of considering the cost-effectiveness of different management strategies when making decisions about resource allocation in healthcare systems.

The present study was the first economic evaluation regarding interventions for sarcopenia management, which was done despite the limitations of the evidence, especially the evidence of the clinical effectiveness of the interventions. Results may be affected by a lack of sufficient evidence for interventions, patients' preference for the treatment, selection bias, and the model design. Expanding and improving scientific evidence regarding the effectiveness of interventions and evidence for patients' acceptance and adherence to treatment in various intervention options can help obtain comprehensive and more accurate results in the future.

## Supplementary Information

**Additional file 1: Figure S1.** Base case cost-effectiveness analysis of sarcopenia management strategies in Iran.

**Additional file 2: Figure S2.** Monte-carlo simulation (Incremental Cost Effectiveness scatter plot) of cost-utility analysis of sarcopenia management strategies.

## Data Availability

This is a secondary study conducted using available evidence and all data and evidence obtained is included in article. The datasets generated and/or analyzed during the current study are not publicly available but are available from the corresponding author on reasonable request.
