# Supplementary material for: Cost-effectiveness analysis of sarcopenia management interventions in Iran
Source: BMC Public Health. 2023 May 4;23:819. doi: 10.1186/s12889-023-15693-w (PMC10157910; doi:10.1186/s12889-023-15693-w)
Supplement: Supplementary file 1 — Additional file 1: Figure S1. Base case cost-effectiveness analysis of sarcopenia management strategies in Iran. [file 12889_2023_15693_MOESM1_ESM.docx]

**Supplementary Information (Fig S1):**


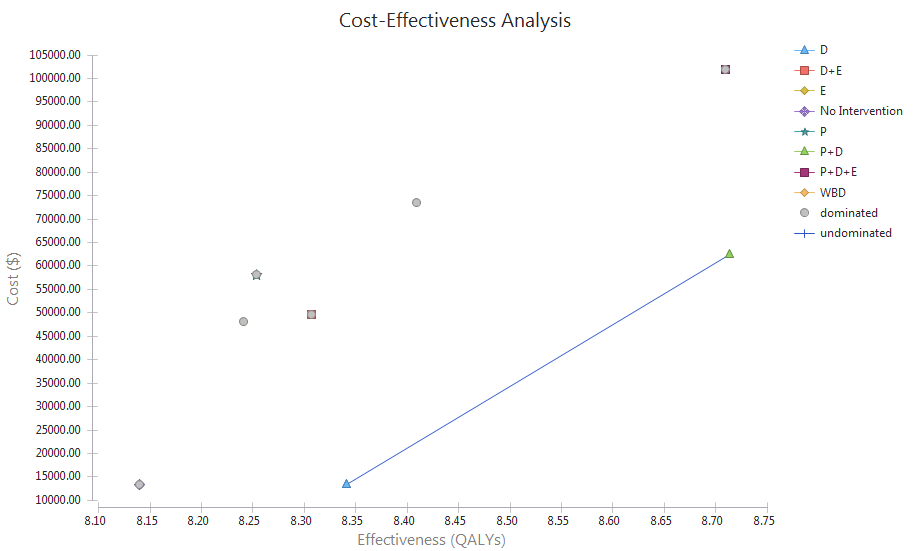


- ***P: Protein; D: Vitamin D_3_; E: Exercise; WBV: Whole body vibration***

**Fig S1: Base case cost-effectiveness Analysis of Sarcopenia Management Strategies in Iran**
