# Supplementary material for: Cost-effectiveness analysis of sarcopenia management interventions in Iran
Source: BMC Public Health. 2023 May 4;23:819. doi: 10.1186/s12889-023-15693-w (PMC10157910; doi:10.1186/s12889-023-15693-w)
Supplement: Supplementary file 2 — Additional file 2: Figure S2. Monte-carlo simulation (Incremental Cost Effectiveness scatter plot) of cost-utility analysis of sarcopenia management strategies. [file 12889_2023_15693_MOESM2_ESM.docx]

**Supplementary Information (Fig S2):**

***
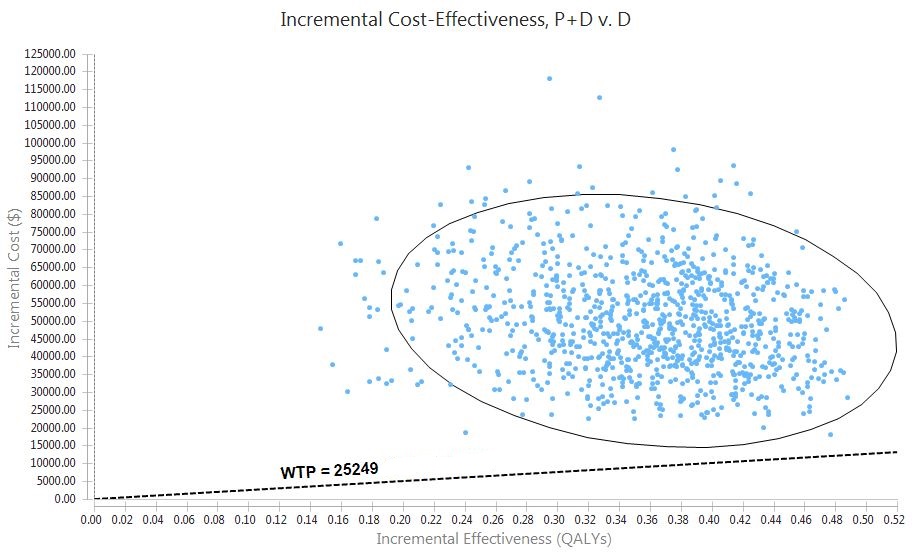
***

- ***P: Protein; D: Vitamin D_3_***

**Fig S2: Monte-carlo Simulation (Incremental Cost Effectiveness scatter plot) of Cost-Utility Analysis of Sarcopenia Management Strategies**
